# Insight Into the Perioperative Management of Type 2 Diabetes

**DOI:** 10.7759/cureus.6878

**Published:** 2020-02-04

**Authors:** Syed Owais Zaidi, Yusra Khan, Bibi S Razak, Bilal Haider Malik

**Affiliations:** 1 Internal Medicine, California Institute of Behavioral Neurosciences and Psychology, Fairfield, USA; 2 Pharmacy, California Institute of Behavioral Neurosciences and Psychology, Fairfield, USA

**Keywords:** perioperative management, diabetes, hyperglycemia, high blood sugars

## Abstract

Diabetic people are at risk of developing acute complications when exposed to stress. Surgery brings a stressful period when the patient is exposed not only to surgical stress but also the effects of medications used during that particular period. The patient’s comorbidities can influence the perioperative management of diabetes. Poorly controlled diabetes can complicate the hospital course.

The literature was searched through PubMed and the articles of the last 5 years, from 2014 to 2019, were looked into. The studies available as a free text, in the English language and related to humans, were included. Inclusion criteria also included adults with type 2 diabetes undergoing surgery.

The perioperative management of diabetes is a challenging one. Apart from the diabetes control; comorbidities, general health, intake, and interaction of medications both anti-diabetic and non-diabetic, type and duration of surgery, are some of the factors that influence the outcome of the surgery. With a variety of options available to manage diabetes currently, it is important to have a good insight into their effects to prevent complications to occur and ensure safe discharge from the hospital. The good control of diabetes is essential in bringing favorable outcomes.

The perioperative management of diabetes should be individualized. Oral anti-hyperglycemic medications, other than sulfonylureas and SGLT2 inhibitors, provide a reasonable alternative to insulin and can be continued safely perioperatively depending upon the type of surgery and the patient is expected to resume oral intake soon postoperatively.

## Introduction and background

The diabetic population is prone to have a complicated hospital course along with the risk of perioperative complications [1]. According to the figure quoted by International Diabetes Federation in 2017, 425 million people between the age range of 18-99, are affected with diabetes and this number is expected to touch 693 million in 2045 [2]. It is essential to review the treatment of diabetes and its complications before undertaking surgery with regard to its effects on fluid and electrolyte balance and cardiovascular system, in terms of interactions with other drugs and the other illnesses like kidney disease, autonomic disorders, coronary artery disease, vascular disease, and high blood pressure [1].

Screening for diabetes is recommended in every patient being planned for surgery [1]. A review of a recent hemoglobin A1c (HbA1c) level before surgery permits risk assessment and attempt to achieve good glycemic control (HbA1c < 69 mmol/l) [3-4]. Hemoglobin A1c (HbA1c) provides an average estimate of blood sugars over the last three months in diabetic patients, thereby not only representing the quality of diabetic control but also permits review and adjustment in treatment to achieve the target, in addition to that, its raised value in some studies indicates a likelihood of early postoperative infection and myocardial infarction [5-6]. 

It has been found in the meta-analysis of studies involving patients with diabetes undergoing surgical procedures only that the blood sugar control in the range of 150-200 mg/dl (8.3-11.1 mmol/l) is associated with decreased perioperative mortality and stroke than a more relaxed target of > 200 mg/dl (11.1 mmol/l). Moreover, strict control between 100 and 150 mg/dl (5.6-8.3 mmol/l) does not result in an added advantage [7]. Surgery in diabetics places them at high risk to develop postoperative infections due to the adverse effects of diabetes on their immune status, wound healing, and blood supply through small vessels [8-10].

Perioperative treatment to achieve good control of diabetes with insulin, either in the form of infusion or bolus has been used over the years due to its rapid action and easy adjustment, however, due to the variability in insulin resistance among the patients, the response is unpredictable and optimal regimen is yet to be known [4, 11-15]. The advent of new treatments in diabetes opens the door of discussion regarding achieving control of diabetes, timing to withhold medications, an adjustment in doses or modification in the treatment, and interactions of the drugs perioperatively. The literature is reviewed to find the answer to these questions.

The search of the literature review is done using the internet and PubMed using the following six regular keywords and three MeSH Words:

Regular keywords

1. Perioperative: It brings the results of 116379.

2. Diabetes: It shows 709582 results.

3. Management: It comes up with the 2830280 results.

4. Complications: It reveals 3190876 results.

5. Insulin: It produces 400938 results.

6. Antidiabetics: It presents with 260645

Regular keywords are summarized in Table 1.

**Table 1:** Regular keywords

KEYWORDS	DATABASE	NUMBER OF RESULTS
Perioperative	PubMed	116379
Diabetes	PubMed	709582
Management	PubMed	2830280
Complications	PubMed	3190876
Insulin	PubMed	400938
Antidiabetics	PubMed	260645

MeSH strategy

The below-mentioned picture emerges by employing MeSH strategy.

1. Perioperative is searched through MeSH and produces six results.

2. Diabetes in MeSH reveals 100 results.

3. Management via MeSH produces 53 results.

MeSH strategy is described in Table 2.

**Table 2:** MeSH strategy

MeSH WORDS	NUMBER OF RESULTS
Perioperative	6
Diabetes	100
Management	53

Inclusion criteria

The following inclusion criteria were used while searching the literature:

1. The last five years of articles were searched from 2014 to 2019.

2. Only free text available was made part of the search.

3. The studies related to humans only were taken into consideration.

4. Adults with type 2 diabetes were included in the review.

5. The review articles and randomized control trials (RCTs) were made part of the review.

6. All the articles were peer reviewed.

Ethical issue

All the data were collected ethically and legally.

Quality assessment tool

No quality assessment tool was applied.

## Review

Perioperative management of diabetes

The common exercise is to hold all oral anti-diabetic medications on the day of undertaking surgery; however, this strategy does not deem fit for those patients undergoing surgery for a shorter period or are expected to resume their diet quickly or are discharged after having a short stay [16-17].

Those patients in whom the diet is withheld for a shorter period, the Association of Anesthetists of Great Britain and Ireland (AAGBI) guideline recommends an individualized approach with the option of carrying on with the antihyperglycemic medications that do not cause low blood sugars and metformin is no exception [18].

A joint Anesthesiology and Diabetology position statement from France also reinforces the idea of sticking with the non-insulin medications in daycare surgeries and only to be stopped in major procedures [19].

Given that, the existence of no definitive strategy to manage blood sugars perioperatively and diverse recommendations from different clinical bodies, it is not unexpected to observe in the audits like National Confidential Enquiry into Patient Outcome and Death (NCEPOD) study, the different approaches being employed in clinical settings to achieve blood glucose targets [20].

Anti-hyperglycemic medications

Metformin

It is the recommended first-line drug to be used in treating type 2 diabetes unless contraindicated [1]. In the past, it is stopped for about 48 hours before surgery because of the overestimated fear of lactic acidosis [21-22]. However, it is prudent to assess the following risk factors before deciding it to continue preoperatively or restart postoperatively [5]:

i. Renal impairment

ii. Use of contrast agents

iii. Dehydration

iv. Severe heart failure (EF < 30%)

Recently, the US Food and Drug Authority (FDA) has permitted it to continue unless the estimated glomerular filtration rate (eGFR) falls below 30 ml/min/1.73m2 [1].

The American Diabetes Association (ADA) proposes holding of metformin on the day of surgery whereas the Association of Anesthetists of Great Britain and Ireland (AAGBI) suggests to continue it with the rest of the oral anti-diabetic medications other than sulfonylureas and sodium-glucose transport inhibitors (SGLTIs) on the day of procedure as it does not cause low blood sugars [16, 23]. It might be safe to restart 48 hours after the major procedure and making sure of the adequate functioning of the kidney [5].

Sulfonylureas

Sulfonylureas have been in the horizon of treatment of diabetes for about 70 years and a usual strategy to withhold it on the day of surgery remains plausible as it is associated with a higher risk of asymptomatic hypoglycemia compare to other antihyperglycemic medications demonstrated in a Continuous Glucose Monitoring (CGM) study [1, 24]. 

A review of meta-analysis showed that high risk is attached with the use of sulfonylureas in causing hospitalization, congestive cardiac failure, and mortality [25].

Sodium-Glucose Co-Transporters 2 (SGLT2) Inhibitors

It is a relatively new class of drugs added to the armamentarium of diabetes management that is associated with good blood glucose control, decreased plasma volume, renal protection, and weight loss [26-28]. It works by facilitating the passage of blood glucose and sodium in the urine irrespective of insulin [29]. However, it is linked with genitourinary infections and diabetic ketoacidosis (DKA) especially euglycemic DKA [1, 30-31]. The occurrence of euglycemic DKA is likely to increase in the peri - and post-operative period if they are not stopped perioperatively or restarted very soon postoperatively [32-35]. The awareness of a risk of developing euglycemic is very important to those who undergo surgery and a very high index of suspicion should be present not to miss it [1]. SGLT2 inhibitors should be restarted postoperatively when the patient looks clinically well and resumes and tolerates his oral intake properly [1].

At present, there is no agreement on the withholding of SGLT2 inhibitors before surgery but the general strategy is to stop them before 24-72 hours or even longer [1, 34-35].

*Dipeptidyl Peptidase 4 (DPP 4) Inhibitors *​​​​​​​

Their use is associated with low risk of hypoglycemia and to stick or discontinue with it perioperatively is unlikely to produce any significant complications and either strategy is acceptable [1].

Glucagon-Like Peptide 1 (GLP-1) Agonists

They are administered through injections and are not linked to produce hypoglycemia instead of nausea and vomiting can occur. Their long - term use is associated with cardiovascular benefit [1, 16, 36-41].

The glucose control is better achieved through GLP - 1 agonist in comparison to insulin as shown in two randomized control studies of cardiac and non-cardiac patients undergoing surgery [16, 42]. However, gastrointestinal adverse effects may become limiting factors to their use [1]. Therefore, either plan of going with or against it perioperatively is workable [1].

*Insulin*​​​​​​​

Perioperative insulin-based management in the form of basal-bolus is quite effective in attaining blood glucose targets than intermittent bolus with rapid-acting insulin in type 2 diabetes [43]. Insulin infusion is also an option and can be used in critical and non-critical situations [1].

Degludec has a long half-life of more than 42 hours and current data are not enough to determine its effect on perioperative blood sugars [1, 5].

The summary of antidiabetic medications used perioperatively is outlined in Table 3.

**Table 3:** Summary of perioperative use of anti-diabetic medications

Summary of Perioperative Use of Anti-Diabetic Medications
Metformin
The American Diabetes Association (ADA) proposes holding of metformin on the day of surgery whereas the Association of Anesthetists of Great Britain and Ireland (AAGBI) suggests to continue it with the rest of the oral anti-diabetic medications other than sulfonylureas and sodium–glucose transport inhibitors on the day of the procedure as it does not cause low blood sugars. It might be safe to restart 48 hours after the major procedure and making sure of the adequate functioning of the kidney
Sulfonylurea
The usual strategy to withhold it on the day of surgery remains plausible
Dipeptidyl peptidase 4 (DPP4) inhibitors
To continue or discontinue it perioperatively is unlikely to produce any significant complications and either strategy is acceptable
Sodium–glucose co-transporters 2 (SGLT2) inhibitors
At present, there is no agreement on the withholding of SGLT2 inhibitors before surgery but the general strategy is to stop them before 24–72 hours or even longer
Glucagon-like peptide 1 (GLP1) agonists
Gastrointestinal adverse effects may become limiting factors to their use. Therefore, either plan of going with or against it perioperatively is workable
Insulin
Perioperative insulin – based management in the form of basal-bolus is quite effective in attaining blood glucose target than intermittent bolus with rapid-acting insulin in type 2 diabetes. Insulin infusion is also an option and can be used in critical and non-critical situations. Degludec has a long half-life of more than 42 hours and current data are not enough to determine its effect on perioperative blood sugars

Assessment of control of blood glucose levels ​​​​​​​

Perioperative assessment of diabetic control is paramount and is evaluated by the following two methods [5]:

i. Hemoglobin A1c (HbA1c)

ii. Blood sugar level

There are several factors causing variation in blood sugar levels, some of them are [5]:

i. Fasting

ii. Stress

iii. Infection

iv. Medications such as steroids

Hemoglobin A1c (HbA1c) Levels

Hemoglobin A1c (HbA1c) level gives a good idea of the blood sugar level over the past three months, thereby helping in the modification of the treatment to obtain the target level [5-6]. Elevated HbA1c links to morbidity, mortality, cardiac injury, and postoperative infection [11]. It might be prudent to postpone the elective procedure when HbA1c level is found > 9% or < 5% to prevent the acute metabolic problems or occurrences of hypoglycemic episodes respectively, perioperatively [5].

Blood Glucose Levels

Blood sugars > 200 mg/dl are associated with morbidity and mortality in a postoperative period [44-46]. The demonstration of a recent dysregulation in blood glucose during perioperative monitoring is not reflected through HbA1c [5].

Hypoglycemia during the perioperative period can cause a significant challenge. In cases of autonomic dysfunction, it is not easy to pick and a close vigilance is required to manage it timely [45]. There are many factors that can predispose to low blood sugars including fasting state, irregular food intake postoperatively, impairment of renal or liver function, interactions with medications like quinolone, beta-blockers, heparin, trimethoprim-sulfamethoxazole, etc [5].

Perioperative risk assessment of type 2 diabetes

Perioperatively, the severity of the following complications of diabetes needs to be assessed:

1. Gastroparesis

2. Heart disease

3. Kidney disease

1. Gastroparesis

It is a quite common complication of long-standing diabetes and may be defined as the slow or delayed emptying of the stomach in the absence of mechanical obstruction [5]. It may present with the following symptoms [47]:

i. Anorexia

ii. Abdominal pain

iii. Nausea

iv. Vomiting

v. Early satiety

vi. Abdominal bloating

It can cause a disturbance in postprandial blood sugars [5]. It poses a risk of aspiration of food contents during anesthesia, therefore, it is important to specifically look for and manage this complication and avoid those medications that can further exacerbate this condition [5].

2. Cardiac Disease

Proper risk stratification of the cardiac disease including coronary artery disease, heart failure, and cardiac autonomic neuropathy should be undertaken through a detailed history, examination, and review of the recent cardiac investigations. High-risk cases should be further evaluated and consulted by the cardiologist.

3. Kidney Disease

Renal evaluation is required to avoid kidney injury postoperatively. Urine albumin to creatinine ratio (ACR) and the estimation of the glomerular filtration rate (eGFR) should be determined before performing major surgery [5]. Nephrotoxic agents should be avoided in high-risk cases.

Perioperative hyperglycemia

High blood sugars in a perioperative period in either group of diabetics or non-diabetics are linked to the increase in the morbidity and mortality postoperatively and therefore are considered an independent risk factor [48-49]. They are associated with the delay in wound healing and postoperative infections [48]. The patient’s outlook is improved if hyperglycemia in the perioperative period is well managed [48]. 

Blood Glucose Targets

Blood sugars above 180 mg/dl are found to be associated with morbidity, especially infections, and mortality [48]. At the same time, there is always a risk of hypoglycemia while attempting control of blood glucose [48]. This risk should be balanced with the cautious approach and the chances of this happening are decreased when blood sugars are targeted to keep in the range from 140-180 mg/dl. However, a wide range between 90-180 mg/dl of blood sugars is relatively easy to target [48].

Recommendations for the Perioperative Management of Blood Glucose

1. Fasting for a longer period should be avoided [48].

2. An attempt should be made to schedule surgery in the morning [48].

3. Blood glucose should be targeted < 180 mg/dl [48].

**Figure 1:**
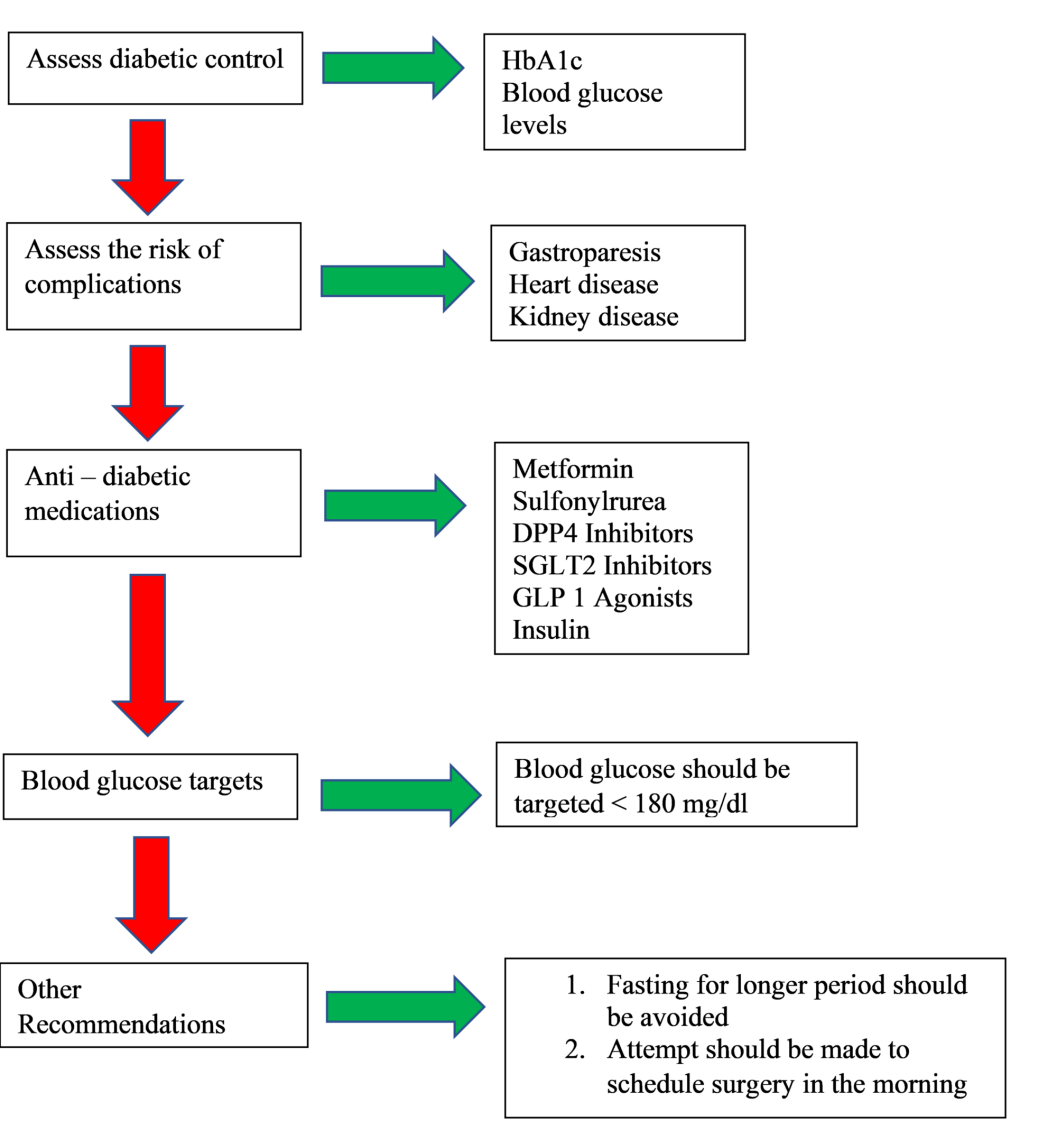
Approach to the Perioperative Management of Diabetes HbA1c:  Hemoglobin A1c; DPP4 Inhibitors:  Dipeptidyl Peptidase 4 Inhibitors; GLP1 Agonists:  Glucagon – Like Peptide 1 Agonists; SGLT2 Inhibitors:  Sodium - Glucose Transporter 2 inhibitors

The approach to perioperative management of diabetes is depicted in Figure 1.

 

Limitations

In this article, the literature was reviewed pertaining to the perioperative management of blood sugars in patients with adult type 2 diabetes who undergo or being planned for surgery. No quality assessment tool has been applied and the articles over the last five years related to humans were selected. The research papers that were in the English language only were chosen and the studies in other languages were not made part of this article. Another limitation was to select those research articles only who were available as a free text on the database of PubMed. The articles with paid subscriptions were not studied.

Table 4 shows the list of articles that were relevant to the review article. 

**Table 4:** Relevant articles

SN	Author’s Name	Year of Publication	Country of Origin of the Study	Title of the Article	Important Points of the Study
1	Deniz Kuzulugil et al [1]	2019	Australia	Recent advances in diabetes treatments and their perioperative implications	Preoperative diabetes management is different from center to center. The convergent approach is required in patients with comorbidities. Sodium glucose transporter 2 inhibitors and sulfonylureas should be withheld before major procedures.
2	Gaëlle Cheisson et al [5]	2018	France	Perioperative management of adult diabetic patients: Preoperative period	Diabetic control should be evaluated by using haemoglobin A1c and Blood glucose levels. Assess for gastroparesis, cardiac and renal status. Management should be individualized depending upon the comorbidities and type of surgery.
3	Gaëlle Cheisson et al [5]	2018	France	Perioperative management of adult diabetic patients: Intraoperative period	High blood sugars > 180 mg/dl peri – operatively carries the risk of morbidity and mortality. Hypoglycemia may occur when strict blood sugars control is attempted.
4	Gaëlle Cheisson et al [5]	2018	France	Perioperative management of adult diabetic patients: Postoperative period	Meticulous monitoring of blood sugars is required to detect and manage hypo and hyperglycemia. Insulin infusion can be replaced with basal – bolus regime. Postoperatively, anti – diabetic medications are prescribed by taking into account previous use of medications, diabetic control, and comorbidities.

## Conclusions

Perioperative management of hyperglycemia is vital to avoid complications and successful outcomes. Blood sugars should be kept from 140 to 180 mg/dl, which is achieved through anti-diabetic medications. The choice of anti-diabetic medications depends on the number of factors including type and duration of surgery, comorbidities of the patients, and estimation of an early resumption of oral intake post surgery. In the usual practice, insulin is used to achieve target blood sugars in a perioperative state affecting the cost and requiring strict monitoring of labs. With the advent of new anti-diabetic medications, does the outlook remain the same or providing other options too? In the daycare settings, or when the patient is expected to be discharged soon, most of the anti-diabetics other than sulfonylureas and SGLT 2 inhibitors, can be continued perioperatively, thus, providing a wide range of choices. The sulfonylureas are associated with a risk of hypoglycemia whereas the SGLT 2 inhibitors are linked to causing diabetic ketoacidosis, especially euglycemic ketoacidosis can be a tricky one to diagnose. The beliefs and fears surrounding metformin are overestimated and it can be used by assessing the overall situation. This emphasizes the individualized approach. The insight into the proper utilization of antihyperglycemic medications other than insulin can boost the confidence and comfort level of both clinicians and patients. However, although there is almost a consensus of keeping blood sugar between 140 and 180 mg/dl perioperatively, there is no universal guideline for the use of anti-diabetic medications in a perioperative state. In the future, this area might require further exploration to come up with more robust and uniform recommendations.
